# Disease Manifestations and Complications in Dutch X-Linked Hypophosphatemia Patients

**DOI:** 10.1007/s00223-023-01172-2

**Published:** 2024-01-16

**Authors:** A. Bosman, N. M. Appelman-Dijkstra, A. M. Boot, M. H. de Borst, A. C. van de Ven, R. T. de Jongh, A. Bökenkamp, J. P. van den Bergh, B. C. J. van der Eerden, M. C. Zillikens

**Affiliations:** 1 Department of Internal Medicine, Erasmus MC, University Medical Center Rotterdam, PO Box 2040, 3000 CA Rotterdam, The Netherlands; 2 Department of Internal Medicine, Leiden University Medical Center, Leiden, The Netherlands; 3 Department of Pediatrics, University Medical Center Groningen, University of Groningen, Groningen, The Netherlands; 4 Department of Internal Medicine, University Medical Center Groningen, Groningen, The Netherlands; 5 Department of Internal Medicine, Radboud University Medical Center, Nijmegen, The Netherlands; 6 Department of Internal Medicine, Amsterdam University Medical Center, Vrije Universiteit, Amsterdam, The Netherlands; 7 Department of Pediatric Nephrology, Amsterdam University Medical Center, Emma Children’s Hospital, Amsterdam, The Netherlands; 8 Department of Internal Medicine, VieCuri Medical Center, Venlo, The Netherlands

**Keywords:** FGF23, Phosphate, Hypophosphatemia, XLH, X-linked hypophosphatemia

## Abstract

X-linked hypophosphatemia (XLH) is the most common monogenetic cause of chronic hypophosphatemia, characterized by rickets and osteomalacia. Disease manifestations and treatment of XLH patients in the Netherlands are currently unknown. Characteristics of XLH patients participating in the Dutch observational registry for genetic hypophosphatemia and acquired renal phosphate wasting were analyzed. Eighty XLH patients, including 29 children, were included. Genetic testing, performed in 78.8% of patients, showed a *PHEX* mutation in 96.8%. Median (range) Z-score for height was − 2.5 (− 5.5; 1.0) in adults and − 1.4 (− 3.7; 1.0) in children. Many patients were overweight or obese: 64.3% of adults and 37.0% of children. All children received XLH-related medication e.g., active vitamin D, phosphate supplementation or burosumab, while 8 adults used no medication. Lower age at start of XLH-related treatment was associated with higher height at inclusion. Hearing loss was reported in 6.9% of children and 31.4% of adults. Knee deformities were observed in 75.0% of all patients and osteoarthritis in 51.0% of adult patients. Nephrocalcinosis was observed in 62.1% of children and 33.3% of adults. Earlier start of XLH-related treatment was associated with higher risk of nephrocalcinosis and detection at younger age. Hyperparathyroidism longer than six months was reported in 37.9% of children and 35.3% of adults. This nationwide study confirms the high prevalence of adiposity, hearing loss, bone deformities, osteoarthritis, nephrocalcinosis and hyperparathyroidism in Dutch XLH patients. Early start of XLH-related treatment appears to be beneficial for longitudinal growth but may increase development of nephrocalcinosis.

## Background

X-linked hypophosphatemia (XLH) is the most common genetic cause of chronic hypophosphatemia. It results from loss-of-function genetic variations in the phosphate-regulating endopeptidase homolog X-linked (PHEX) gene, leading to increased circulating fibroblast growth factor 23 (FGF23) levels [1–3]. Excess FGF23 levels can cause hypophosphatemia by reducing renal phosphate reabsorption and decreasing 1,25-dihydroxy-vitamin D (1,25(OH)_2_D) production [4, 5]. Chronic hypophosphatemia can cause multiple problems including muscle weakness and decreased bone mineralisation. XLH is characterized not only by rickets in children and osteomalacia in adults, but also bone pains, increased risk of (pseudo)fractures, dental and hearing problems, osteoarthritis and enthesopathies [6]. Prevalence has been estimated between 1:20,000 and 60,000 children [7–9]. Conventional treatment consists of active vitamin D and oral phosphate supplementation but is associated with the development of nephrocalcinosis/nephrolithiasis and hyperparathyroidism [6]. The anti-FGF23 antibody burosumab was approved for use in children with XLH in the Netherlands in 2018 and for adults in 2020. The observational registry for genetic hypophosphatemia and acquired renal phosphate wasting in The Netherlands (ORPHOS-NED) has been set up to evaluate retrospectively and prospectively demographic, biochemical, radiological and genetic characteristics, treatment and quality of life of patients with genetic and acquired forms of chronic hypophosphatemia due to renal phosphate wasting. In the current study, we describe for the first time the clinical characteristics of XLH patients in the Netherlands and occurrence of complications including (pseudo)fractures, nephrocalcinosis/nephrolithiasis, and hyperparathyroidism.

## Methods

### Population

ORPHOS-NED is an ongoing nationwide observational cohort study on chronic hypophosphatemia, which started inclusion in 2020. Currently, 9 hospitals (academic and non-academic) participate. Adult and pediatric nephrologists and endocrinologists were contacted to identify patients with chronic hypophosphatemia. Patients of all ages with the diagnosis of hypophosphatemic rickets/osteomalacia based on clinical, radiological, biochemical and/or genetic results were included. In addition, patients with chronic hypophosphatemia defined as a serum phosphate level below the lower limit of normal of the reference range in adults and below the age-dependent reference value in patients younger than 18 years of age in two consecutive blood samples taken at least three weeks apart were also included. Patients with chronic hypophosphatemia due to a known dietary phosphate deficiency or malabsorption, primary hyperparathyroidism or due to a general proximal renal tubulopathy were excluded, as well as patients with hypophosphatemia in acute clinical settings such as refeeding. After informed consent, retrospective data from the first clinical presentation and onwards was collected from medical files. Data in the registry has been—and will be—updated yearly. In the current study, we analyzed the data of adults and children with a diagnosis of XLH who were included in ORPHOS-NED before November 1st 2022. The diagnosis was either confirmed based on the finding of a (likely) pathogenic genetic variation in *PHEX* or suspected based on the phenotype and/or family history. Most pediatric XLH patients in ORPHOS-NED were included at the University Medical Center Groningen, which is the Dutch reference center for burosumab treatment in children.

### Patient Characteristics

We analyzed the following parameters: clinical characteristics at inclusion [age, sex, family history, height and weight, body mass index (BMI)], genetic testing, medical treatment at time of inclusion, biochemical findings at inclusion (including serum phosphate, calcium, parathyroid hormone (PTH), FGF23, 25-hydroxy-vitamin D (25OHD), 1,25(OH)_2_D, alkaline phosphatase (ALP), *N*-terminal propeptide of type I procollagen (P1NP), bone-specific alkaline phosphatase (BALP), *C*-terminal telopeptide of type I collagen (CTX), ratio of tubular maximum reabsorption of phosphate to glomerular filtration rate (TmP/GFR)) and historical data on renal imaging (nephrocalcinosis, nephrolithiasis), bone deformities, osteoarthritis, orthopedic interventions, fractures, hyperparathyroidism and hearing loss.

To compare height in XLH patients to the general population in the Netherlands, we calculated age- and sex-specific Z-scores [10]. If height and/or weight in adults were not measured in the year of study inclusion, we collected these data reported within 5 years before inclusion. BMI (kg/m^2^) was calculated using weight and height. For children, we calculated age- and sex-specific Z-scores for BMI using data from the Fifth National Growth study [11]. In adults, healthy weight, overweight and obesity were defined as a BMI between 18.5–25 and 25–30 and greater or equal to 30, respectively. In children, we used age- and sex-specific cut-off values for BMI defined by the Dutch center for youth healthcare [12].

Serum phosphate concentrations are higher in infants than adults, and gradually decrease during childhood and adolescence. To analyze serum phosphate in the total pediatric population, we calculated Z-scores by applying age-related formulas for mean and standard deviation constructed by Verploegen et al. [13]. Hypophosphatemia was defined as Z-score below -2. The eGFR was calculated using the Chronic Kidney Disease Epidemiology Collaboration equation in adults and the Chronic Kidney Disease in Children Under 25 equation in children [14, 15]. TmP/GFR was calculated whenever there was simultaneous measurement of phosphate and creatinine in serum and in a urine portion, irrespective of XLH-related treatment. TmP/GFR was calculated using the formula provided by Barth et al. and the age-related reference ranges for TmP/GFR as calculated by Payne et al. were applied [16, 17].

Currently, FGF23 concentrations in the Netherlands are measured by a *C*-terminal FGF23 (cFGF23) assay (Immutopics, San Clemente, CA, USA) at Amsterdam University Medical Center (upper limit of normal: 125 RU/mL) [18]. This assay measures both intact and *C*-terminal FGF23 [19]. We only analyzed FGF23 concentrations that were measured by this assay. Burosumab treatment can greatly increase FGF23 concentrations [20]. Therefore, patients with FGF23 concentrations measured under burosumab treatment were excluded from analyses on FGF23.

In adults, we compared values for the bone formation markers BALP and P1NP and the bone resorption marker CTX with the age- and sex-specific upper limit of normal. In children, we calculated age- and sex-specific Z-scores. Z-scores above 2 were considered increased.

Hypercalciuria from 24 h urinary calcium excretion [21] is defined in the Netherlands as excretion above 7.5 mmol (300 mg) per day. As collection of 24 h urine is challenging in children and therefore often not performed, we used the calcium/creatinine ratio in urine samples taken in the year prior to the diagnosis of nephrocalcinosis [21]. We compared these values to age-related 95th percentile reference values as defined by the Dutch Society for Pediatric Nephrology [22].

Reports of hearing loss were collected from the medical records. Data on osteoarthritis were collected from imaging. Data on bone deformities were collected from physical examinations and from imaging. Data on fractures include all fractures ever sustained as reported in the medical history and/or on imaging. Due to the study design, it was not possible to distinguish between fractures and pseudofractures. Data on orthopedic interventions include all orthopedic interventions documented in the medical records. Data on nephrocalcinosis and nephrolithiasis were collected from renal imaging and from medical history.

### Statistical Analyses

Demographic characteristics are summarized by standard descriptive summaries (e.g., medians and (interquartile) ranges for continuous variables and percentages for categorical variables). The Shapiro–Wilk test was used to assess normality. Associations between categorical variables were assessed with a Chi square test or Fisher’s exact test. Differences in medians between groups were tested using Mann–Whitney *U* Test. Correlations were analyzed using Spearman’s Rank and Kendal’s tau b. Correlation analyses were performed between age at start of XLH-related medical treatment and height Z-score at inclusion, age of the first orthopedic intervention, occurrence of nephrocalcinosis and the age it was observed for the first time. In addition, correlations were analyzed between serum phosphate at inclusion and simultaneously measured serum 25(OH)D, 1,25(OH)_2_D, PTH, and cFGF23; and between ALP in adults and serum phosphate, 25(OH)D, 1,25(OH)_2_D and the bone markers P1NP and CTX.

Study parameters were analyzed in all patients. In case of missing data, we report the results from analyses in the study population with complete data.

## Results

### XLH Patient Cohort

**Fig. 1:**
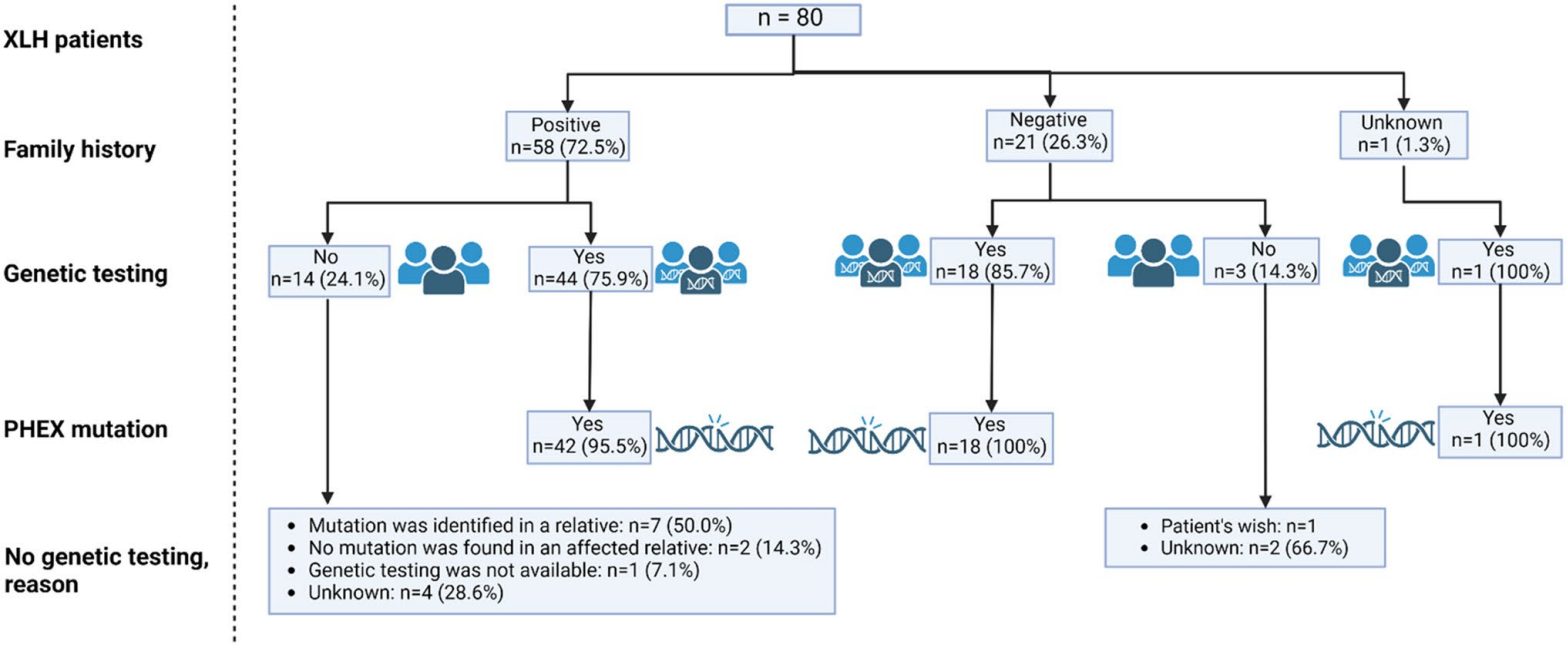
Family history and genetic testing in Dutch XLH patients

On November 1st 2022, ORPHOS-NED included 141 patients with chronic hypophosphatemia of whom 80 patients from 7 academic centers were diagnosed with XLH: 29 children and 51 adults. Data on general demographic characteristics of these 80 patients, including sex, height, etc. are shown in Table 1. In adults with a BMI measurement available, 32.1% was overweight (BMI between 25 and 30 kg/m^2^), while 32.1% was obese (BMI above 30 kg/m^2^). When considering age- and sex-specific cut-off values for BMI in children, 25.9% was overweight and 11.1% obese. Genetic testing, using Sanger sequencing of *PHEX* or the next generation sequencing panels for hypophosphatemia, was performed in 63 patients (78.8%) and showed in 61 (96.8%) a (likely) pathogenic variation in *PHEX* while in 2 patients no mutation was found (Fig. 1). The majority of patients (*N* = 58, 72.5%) had a positive family history for hypophosphatemia, bone deformities or short stature. Out of 58 patients with a positive family history, 32 (55.2%) had an affected mother and 9 (15.5%) had an affected father. Maternal grandfathers and grandmothers were affected in 6 (10.3%) patients, while a paternal grandfather was affected in 1 (1.7%) patient and paternal grandmothers were affected in 26 (44.8%) patients.

**Table 1:** Demographic characteristics of Dutch XLH patients at inclusion in ORPHOs-NED

	Children *N* = 29	Adults *N* = 51
Age, years	9.7 (1.6 to 17.4)	45.8 (19.5 to 76.4)
Female, *n*(%)	13 (44.8%)	36 (70.6%)
Final height, cm		
Females	–	155 (136 to 177)
Males	–	172 (157 to 184)
Height (Z-score) at inclusion	− 1.4 (− 3.7 to 1.0) [27]	− 2.5 (− 5.5 to 1.0) [45]
Males	− 1.4 (− 3.5 to 0.1) [16]	− 1.8 (− 3.8 to 0.03) [13]
Females	− 1.6 (− 3.7 to 1.0) [11]	− 2.5 (− 5.5 to 1.0) [32]
Body mass index, Z-score	1.2 (− 1.4 to 3.5) [27]	–
Males	1.3 (− 1.4 to 3.5)	–
Females	0.4 (0.0 to 2.7) [11]	–
Body mass index, kg/m^2^	–	27.1 (19.5 to 44.0) [28]
Males	–	28.7 (23.6 to 34.0) [8]
Females	–	25.9 (19.5 to 44.0) [20]
Adiposity, *n*(%)^a^		
Underweight	1 (3.7%)	–
Healthy	16 (59.3%)	10 (35.7%)
Overweight	7 (25.9%)	9 (32.1%)
Obesity	3 (11.1%)	9 (32.1%)

Continuous values are displayed as median (range), categorical variables are displayed in absolute counts (%). [*n*] indicates the number of patients in whom these parameters were available

*BMI* body mass index

^a^In adults, healthy weight, overweight and obesity were defined as a BMI between 18.5–25, 25–30, and ≥ 30, respectively. In children, age- and sex-specific cut-off values for BMI were applied

### Medication Use

In adults, 51.0% (*n *= 26) received active vitamin D monotherapy, while 31.4% (*n* = 16) was treated with a combination of active vitamin D and phosphate supplements. One patient received phosphate supplementation without active vitamin D and eight patients were not using XLH-related medication, in the year of inclusion. All children used either active vitamin D, phosphate supplementation or burosumab in the year of inclusion: 58.6% (*n* = 17) received a combination of active vitamin D and phosphate supplementation, while 79.3% (*n* = 23) used burosumab, indicating that some children were switched from conventional therapy to burosumab during the year of inclusion.

The age at start of XLH-related medical treatment could be traced back in 79.3% of children (*n* = 23) and in 78.4% of adults (*n* = 40). Median age at start was 2.8 years in children (range: 0.3 to 13.3) and 6.9 years in adults (range: 0.0 to 43.3). Only 1 adult patient had never received XLH-related medical treatment. Younger age at start of XLH-related treatment was associated with higher height Z-score at inclusion (Spearman’s rho: − 0.33, *P* = 0.013).

### Laboratory Measurements

Table 2 depicts the results from laboratory measurements at the time of inclusion. Hypophosphatemia was present in 93.1% of children and in 86.0% of adults. TmP/GFR was below the age-related lower limit of normal in 92.6% of children. Median (range) *C*-terminal FGF23 (cFGF23) was 189 (82; 517) RU/mL in children and 109 (58; 1091) RU/mL in adults. One adult patient had a cFGF23 concentration of 1091 RU/mL with a serum phosphate concentration of 0.6 mmol/L and an eGFR of 87.9 ml/min/1.73 m^2^.

**Table 2:** Biochemical characteristics of Dutch XLH patients at inclusion in ORPHOS-NED

	Children *N* = 29	Adults *N* = 51
Phosphate, mmol/L	0.86 (0.21)	0.66 (0.19) [50]
Hypophosphatemia, *n*(%)		43 (86.0%)
Phosphate Z-score	− 3.7 (1.6)	
Hypophosphatemia (< -2SD), n(%)	27 (93.1%)	
Calcium, mmol/L	2.37 (0.13)	2.41 (0.17) [50]
Potassium, mmol/L	4.1 (0.6) [12]	4.1 (0.5) [39]
Sodium, mmol/L	140 (1.8) [12]	140 (2.0) [37]
Magnesium, mmol/L	0.80 (0.07) [23]	0.79 (0.08) [24]
Alkaline phosphatase, U/L	398 (214)	95 (44) [45]
Increased, *n*(%)	13 (44.8%)	20 (44.4%)
PTH		
Normal, *n*(%)	15 (51.7%)	32 (74.4%)
Increased, *n*(%)	7 (24.1%)	9 (20.9%)
Unknown, *n*(%)	7 (24.1%)	2 (4.7%)
Missing, *n*(%)	–	8 (15.7%)
25(OH)D, nmol/L	71.5 (33.3) [14]	71.5 (33.3) [30]
Deficient, *n*(%)	2 (14.3%)	7 (23.3%)
1,25(OH)_2_D, pmol/L	166.5 (100.5) [24]	79.0 (39.5) [5]
Low, *n*(%)	1 (4.2%)	–
High, *n*(%)	8 (33.3%)	–
cFGF23, RU/mL	189 (191) [13]	109 (97) [7]
Increased, *n*(%)	11 (84.6%)	3 (42.9%)
Creatinine, µmol/L	38.0 (13.0) [28]	59.0 (17.0) [50]
eGFR, ml/min/1.73 m^2 a^	123.3 (38.5) [26]	105.4 (27.1) [50]
< 60 ml/min/1.73 m^2^, *n*(%)		2 (4.0%)
P1NP, ng/mL	817.5 (222.5) [10]	69.5 (43.3) [26]
Increased, *n*(%)		13 (50.0%)
P1NP, Z-scores	1.22 (1.44) [8]	–
> 2SD, *n*(%)	2 (25.0%)	
Ctx, µg/L	0.18 (0.14) [10]	0.43 (0.30) [25]
Increased, *n*(%)		8 (32.0%)
CTx, Z-scores	2.7 (3.4) [8]	–
> 2SD, *n*(%)	5 (62.5%)	
BALP, U/L	189.1 (62.7) [10]	32.0 [1]
BALP, Z-scores	4.4 (3.6) [9]	–
> 2SD, *n*(%)	8 (88.9%)	
TmP/GFR, mmol/L; median(range)	0.69 (0.22 to 1.48) [27]	0.49 (0.18 to 0.58) [3]
< Age-related reference range^b^, *n*(%)	25 (92.3%)	3 (100%)
24 h urine calcium, mmol/24 h; median(range)	3.0 (2.2 to 3.8) [2]	3.4 (0.6 to 14.5) [34]
Calcium/creatinine ratio, mmol/mmol (urine portion); median(range)	0.83 (0.20 to 6.46) [29]	1.13 (0.96–4.90) [4]

Continuous values are displayed as median (interquartile range) unless otherwise stated, categorical variables are displayed in absolute counts (%). [*n*] indicates the number of patients in whom these parameters were available

*25(OH)D* 25-hydroxyvitamin-D, *1,25(OH)*_*2*_*D* 1,25-dihydroxy-vitamin D, *BALP* bone-specific alkaline phosphatase, *cFGF23 C*-terminal fibroblast growth factor 23, *CTx C*-terminal telopeptide of type I collagen, *eGFR* estimated glomerular filtration rate, *P1NP N*-terminal propeptide of type 1 procollagen, *PTH* parathyroid hormone, *TmP/GFR* ratio of tubular maximum reabsorption of phosphate to glomerular filtration rate

^a^eGFR was calculated in adults based on the Chronic Kidney Disease Epidemiology Collaboration and in children based on the Chronic Kidney Disease in Children Under 25 equation

^b^The age-related reference ranges for TmP/GFR were derived from Payne et al. [17]

At inclusion, PTH was increased in 24.1% of children and in 20.9% of adults. Among the patients with increased PTH, all children had normal serum calcium concentrations, while 1 adult patient had hypercalcemia and 1 adult patient had hypocalcemia. In addition, 14.3% of all children had a 25(OH)D concentration below 50 nmol/L, compared to 23.3% of adults. Serum 1,25(OH)_2_D was measured in almost all children with a median (IQR) of 166.5 (100.5) pmol/L. ALP was increased in 44.8% of children and 44.4% of adults.

In children, phosphate Z-score was inversely correlated with serum 25(OH)D concentrations (Spearman’s rho: − 0.622, *P* = 0.018, *n *= 14). Phosphate Z-score was not correlated with serum 1,25(OH)_2_D, serum cFGF23 or increased PTH. In adults, serum ALP inversely correlated with serum 25(OH)D (Spearman’s rho: − 0.474, *P* = 0.008, *n* = 30), but not with serum 1,25(OH)_2_D. Serum phosphate inversely correlated with serum *C*-terminal FGF23 (Spearman’s rho: − 0.766, *P* = 0.027, *n* = 8) but not with serum 25(OH)D, 1,25(OH)_2_D, PTH or ALP.

In adults, serum ALP positively correlated with serum P1NP (Spearman’s rho: 0.731, *P* < 0.001, *n* = 26) and with serum CTX concentrations (Spearman’s rho: 0.614, *P* = 0.001, *n* = 25).

### Hearing Loss, Bone Deformities and Osteoarthritis

Hearing loss was reported in 6.9% of children and in 31.4% of adults. In addition, 11.8% of adults used hearing aids. Results on the age at onset of hearing loss and audiometry are shown in Table 3. Data on occurrence of bone deformities and osteoarthritis are shown in Table 4. The majority of patients (75.0%), had developed a deformity of the knee (53.8% *genu varum* and 31.3% *genu valgum*). In addition, bowing of femora or of lower legs was present in 20% and 11.3%, respectively. Hip deformities were observed in 12.5% of patients and spinal deformities, including scoliosis and increased lumbar lordosis in 7.5% of patients.

**Table 3:** Hearing loss in children and adults with XLH in the Netherlands

	Total *N* = 80	Children *N* = 29	Adults *N* = 51
Patient reported hearing loss, *n*(%)	18 (22.5%)	2 (6.9%)	16 (31.4%)
Hearing loss by audiometry, *n*(%)	10 (12.5%)	3 (10.3%)	7 (13.7%)
Use of hearing aids, *n*(%)	6 (7.5%)	–	6 (11.8%)
Age at (reported) hearing loss	35.5 (5.0 to 69.8)	11.5 (8.3 to 14.8)	36.8 (5.0 to 69.8) [15]

Continuous values are displayed as median (range) unless otherwise stated, categorical variables are displayed in absolute counts (%)

*n* indicates the number of patients in whom these parameters were available

**Table 4:** Bone- and joint characteristics in children and adults with XLH in the Netherlands

	Total *N* = 80	Children *N* = 29	Adults *N* = 51
Bone deformities			
Deformity of the spine, *n*(%)	6 (7.5%)	1 (3.4%)	5 (9.8%)
Deformity of the hips	10 (12.5%)	2 (6.9%)	8 (15.7%)
Bowing of the femora, *n*(%)	16 (20.0%)	5 (17.2%)	11 (21.6%)
Deformity of the knee, *n*(%)	60 (75.0%)	25 (86.2%)	35 (68.6%)
Genu varum	43 (53.8%)	13 (44.8%)	30 (58.8%)
Genu valgum	25 (31.3%)	15 (51.7%)	10 (19.6%)
Bowing of the lower legs, *n*(%)	9 (11.3%)	5 (17.2%)	4 (7.8%)
Osteoarthritis			
Osteoarthritis of any joint, *n*(%)	26 (32.5%)	–	26 (51.0%)
Osteoarthritis of the hip, *n*(%)	15 (18.8%)	–	15 (29.4%)
Osteoarthritis of the knee, *n*(%)	13 (16.3%)	–	13 (25.5%)
(pseudo)Fractures			
History of a *(pseudo)*fracture, *n*(%)	18 (22.5%)	3 (10.3%)	15 (29.4%)
Age of first *(pseudo*)fracture	24.0 (5.3 to 54.0)	6.5 (5.3 to 7.0)	34.7 (11.8 to 54.0)
Number of *(pseudo)*fractures	1.5 (1.0 to 14.0)	1.0 (1.0 to 1.0)	2.0 (1.0 to 14.0)
*(pseudo)*Fracture location, *n*(%)			
Vertebrae	1 (5.6%)	–	1 (6.7%)
Upper arm	1 (5.6%)	1 (33.3%)	–
Hand/wrist	2 (11.1%)	–	2 (13.3%)
Rib	2 (11.1%)	–	2 (13.3%)
Upper leg	4 (22.2%)	1 (33.3%)	3 (20.0%)
Lower leg	3 (16.7%)	–	3 (20.0%)
Foot	8 (44.4%)	1 (33.3%)	7 (46.7%)

Continuous values are displayed as median (range), categorical variables are displayed in absolute counts (%)

### Fractures and Orthopedic Interventions

A fracture history was reported in 22.5% of the total population and in 29.4% of the adult population. Data on age at time of the first (pseudo)fracture and their number and location are shown in Table 4. In addition, 43.8% (*n* = 35) of patients had undergone at least one orthopedic intervention, which was performed before the age of 18 years in the majority (68.6%). Data on number and type of orthopedic interventions and age at time of the first orthopedic intervention are shown in Table 5. The age at initiation of XLH-related medication did not differ between patients who sustained a (pseudo)fracture or underwent an orthopedic intervention and patients who did not. There was a significant correlation between age at initiation of XLH-related medication and age at the *first* orthopedic intervention (Spearman’s rho: 0.413, *P* = 0.029).

**Table 5:** Orthopedic interventions in children and adults with XLH in the Netherlands

	Total *N* = 80	Children *N* = 29	Adults *N* = 51
History of orthopedic intervention, *n*(%)	35 (43.8%)	10 (34.4%)	25 (42.4%)
Age of first orthopedic intervention	13.5 (1.5 to 58.5)	5.8 (1.5 to 13.5)	16.7 (2.3 to 58.5)
Number of orthopedic interventions	2.0 (1.0 to 10.0)	2.0 (1.0 to 7.0)	3.0 (1.0 to 10.0)
Procedure			
Osteotomy, *n*(%)	21 (60.0%)	1 (10.0%)	20 (80.0%)
Guided growth, *n*(%)	7 (20.0%)	5 (50.0%)	2 (8.0%)
Fracture fixation, *n*(%)	3 (8.6%)	1 (10.0%)	2 (8.0%)
Joint replacement, *n*(%)	7 (20.0%)	–	7 (28.0%)

Continuous values are displayed as median (range) unless otherwise stated, categorical variables are displayed in absolute counts (%)

### Nephrocalcinosis, Nephrolithiasis and Hyperparathyroidism

About half of patients with renal imaging data had nephrocalcinosis and 9.7% had nephrolithiasis. Data on patients’ age at time of discovery of nephrocalcinosis and duration of XLH-related medical treatment before discovery of nephrocalcinosis are shown in Table 6. Of the children with nephrocalcinosis and available urine samples with calcium and creatinine measurements taken the year before or at time of the nephrocalcinosis diagnosis, none had a ratio of calcium-creatinine above the age-related 95th percentile reference value. Three out of the five adults with 24 h urine calcium measurements at the time of or in the year before the diagnosis of nephrocalcinosis had hypercalciuria. The age at start of XLH-related medication was inversely correlated with occurrence of nephrocalcinosis (Kendal’s tau b: − 0.298, *P* = 0.007) and positively correlated with age at diagnosis of nephrocalcinosis (Spearman’s rho: 0.468, *P* = 0.011). Results were similar when restricting to patients who started with conventional treatment. Patients who started treatment before the age of 2 years developed nephrocalcinosis more often than patients who started treatment thereafter, 66.7% (10/15) versus 33.3% (14/42) (*P* = 0.035). The time between start of XLH-related medical treatment and discovery of nephrocalcinosis was not significantly different between these two groups (*P* = 0.709).

**Table 6:** The prevalence of nephrocalcinosis in children and adults with XLH in the Netherlands

	Total *N* = 80	Children *N* = 29	Adults *N* = 51
Data on renal imaging, *n*(%)	72 (90.0%)	29 (100.0%)	43 (84.3%)
Nephrocalcinosis			
Nephrocalcinosis, *n*(%)	35 (47.2%)	18 (62.1%)	17 (33.3%)
Patients’ age at diagnosis of nephrocalcinosis, median (range)	9.5 (1.0 to 72.5)	5.5 (1.0 to 15.0)	17.8 (8.0 to 72.5)
Duration of XLH-related medical treatment before discovery of nephrocalcinosis, years, median (range)	4.9 (0.3 to 69.3) [24]	1.7 (0.3 to 14.8) [13]	12.5 (4.0 to 69.3) [11]
Nephrolithiasis			
Nephrolithiasis, *n*(%)	7 (9.7%)	2 (6.9%)	5 (11.6%)
Patients’ age at diagnosis of nephrolithiasis, median (range)	20.7 (11.5 to 55.3)	13.6 (11.5 to 15.8)	51.7 (14.8 to 55.3)

Continuous values are displayed as median (range) unless otherwise stated, categorical variables are displayed in absolute counts (%)

*n* indicates the number of patients in whom these parameters were available

The lifetime prevalence of increased PTH concentrations was 75.9% in the pediatric population and 54.9% in adults (Table 7). A period of hyperparathyroidism for more than six months was present in 37.9% (11/29) of the pediatric and in 35.3% (18/51) of the adult population. Concurrent hypercalcemia was observed in 1/11 child and in 7/18 adults. 11.1% (2/18) of adults with prolonged hyperparathyroidism had received calcimimetics, while 22.2% (4/18) of the total group of adults with prolonged hyperparathyroidism and 57.1% (4/7) of adults with hyperparathyroidism with hypercalcemia had undergone total or subtotal parathyroidectomy.

**Table 7:** The lifetime prevalence of hyperparathyroidism in children and adults with XLH in the Netherlands

	Total *N* = 80	Children *N* = 29	Adults *N* = 51
Occurrence of hyperparathyroidism, *n*(%)	50 (62.5%)	22 (75.9%)	28 (54.9%)
With normal serum calcium, *n*(%)	40 (80.0%)	15 (68.2%)	25 (89.3%)
Occurrence of hyperparathyroidism > 6 months, *n*(%)		11 37.9%)	18 (35.3%)
Hyperparathyroidism with hypercalcemia, *n*(%)		1 (9.1%)	7 (38.9%)
Parathyroidectomy, *n*(%)		–	4 (22.2%)

## Discussion

This description of 80 Dutch XLH patients included in ORPHOS-NED provides insight in the manifestations, complications and treatment of this debilitating disease in children and adults in the Netherlands. This study confirms the high prevalence of adiposity, hearing loss, bone deformities, osteoarthritis, nephrocalcinosis and hyperparathyroidism. Early start of XLH-related treatment appears to be beneficial for longitudinal growth but may be harmful for the development of nephrocalcinosis. ORPHOS-NED is an ongoing study but has already been set up in all academic hospitals in The Netherlands and includes a comprehensive review of all paper and electronic medical files of the participants. This approach enables us to provide a detailed description of this patient population and compare standard of care in the Netherlands to international guidelines.

The majority of patients in our study, 72.5%, had a positive family history for hypophosphatemia or a bone related condition, in agreement with a cohort study conducted by Rafaelsen et al. who reported a positive family history in 78.6% of pediatric patients with hereditary hypophosphatemia [9]. Still, a considerable number of cases will be sporadic, indicating that XLH should not be excluded based on a negative family history. Decreased growth rate in children is one of the characteristic features of XLH. Beck-Nielsen et al*.* reported a mean Z-score of − 1.9 in a cohort of patients with hypophosphatemic rickets, including XLH [23]. Likewise, height at inclusion was decreased in our cohort with a median Z-score of − 1.4 in children and − 2.5 in adults. We found that age at start of XLH-related medical treatment was inversely correlated with the height Z-score at inclusion. This finding is in agreement with previous studies that showed that early initiation of treatment is beneficial for the growth rate of children [24–26]. This study and previous studies found that adiposity as measured by BMI is prevalent in XLH patients, both in children and in adults [27, 28]. Moreover, obesity was more prevalent in this population of children and adults with XLH than in the general Dutch population [29]. Unfavorably, a higher BMI is causally associated with lower serum phosphate in the general population, and negatively effects gait and the lateral trunk lean in XLH (known as waddling gait) [28, 30]. BMI may not be a suitable parameter for defining adiposity in XLH patients with short stature and bone deformities but a recent study in pediatric XLH patients showed increased body fat percentage and decreased muscle mass measured by bioelectrical impedance analysis [31]. Prevention and treatment of obesity should therefore be part of clinical care [6]. It should be noted that there was some missing data of BMI in adults which could cause bias because patients who are overweight may have their weight followed more closely than patients with a BMI in the normal range.

Concerning laboratory measurements, we found that the bone resorption marker CTX and the bone formation marker P1NP were increased in 32.0% and 50.0% of adults, respectively, indicating increased bone turnover. This is similar to what was recently reported by Hansen et al. [32] In addition, we found that ALP was highly correlated with beta CTX and P1NP in adults. ALP is already used as a marker of osteoblast activity and degree of rickets in XLH [33, 34]. Our finding suggests that ALP may be useful in clinical practice as an indicator for increased bone turnover in adult XLH patients. As expected, hypophosphatemia was highly prevalent in our cohort and serum phosphate inversely correlated with cFGF23 in adults. There was no correlation between serum phosphate and cFGF23 in children, possibly because they were on either conventional treatment or burosumab, which may have obscured any existing correlations. In addition, the cFGF23 concentrations at inclusion both in children and adults are much lower than reported in other FGF23-mediated hypophosphatemic disorders such as tumor-induced osteomalacia [35].

Hearing difficulties are one of the medical problems that have been associated with XLH [36]. A study in mice showed that hearing problems may be caused by hypomineralization of the ossicles but other pathways have also been suggested [37, 38]. In this study, subjective hearing loss was reported by almost one third of adults and also in 6.9% of children. Data on pure tone audiometry were available for only a few patients. Still, subjective hearing loss was less prevalent than reported by Davies et al., who found a prevalence of 48% [39]. Notably, pure tone audiometry detected hearing loss in 76% of subjects in the same study. Taken together, these data show that hearing difficulties are a prevalent, but underreported feature of XLH and that audiometry should be performed routinely in this patient population.

Rickets in childhood can cause bone deformities, which may necessitate orthopedic interventions. Moreover, XLH patients often develop early onset osteoarthritis which may also require orthopedic interventions. Bowing of knees was reported in 75% of the total population, with *genu varum* being more prevalent than *genu valgum*. This is in agreement with recent studies in adult XLH patients by Orlando et al. and Mindler et al. [28, 40]. Osteoarthritis of the hip and knee was reported in about a quarter of adult patients, similar to a cohort study of FGF23-mediated hypophosphatemic rickets by Beck-Nielsen et al. but much lower than reported in other previous studies [23, 28, 41]. Our retrospective study design is prone to selection bias because extensive radiographic imaging will only have been performed in symptomatic patients. Similarly, Mindler et al. studied patients with availability of full length radiographs and Skrinar et al. conducted a burden of disease survey among adults with XLH, including questions on presence of osteoarthritis [28, 41]. To accurately examine the prevalence of osteoarthritis in XLH, future prospective studies with standardized imaging are essential.

Enthesopathies in XLH, including bone proliferations at ligament attachments or calcification of ligaments are also associated with reduced quality of life [42]. Unfortunately, enthesopathies were not routinely assessed by radiologists in our study population, refraining us from drawing conclusions on their prevalence.

Almost half of our population had undergone one or more orthopedic interventions with osteotomies being the most frequent, in agreement with a previous study by Chesner et al. but less than reported in the burden of disease survey by Skrinar et al. [41, 43]. Moreover, almost 30% of adult XLH patients had sustained at least one fracture, mainly in legs and feet, which is less than reported by Javaid et al. who analyzed subjects of a clinical trial and participants in an online survey. In contrast, Beck-Nielsen et al. reported a fracture rate of 18% in patients with hypophosphatemic rickets and even a reduced fracture risk compared to controls [1, 23]. It should be noted that we did not distinguish between fractures and pseudofractures or Looser’s zones.

In a consensus statement, Haffner et al. recommend to screen for nephrocalcinosis by performing regular renal ultrasonographies [6]. Nephrocalcinosis is a well-known complication of conventional medical XLH treatment, thought to be the result of medication-induced hypercalciuria. Previous studies also report associations between oral phosphate use and nephrocalcinosis risk but not with use and duration of active vitamin D [44, 45]. Ninety percent of our population had undergone renal imaging and nephrocalcinosis was detected in 62% of children and 33% of adults. The prevalence of nephrocalcinosis in our pediatric population is higher than reported recently in a Norwegian cohort of XLH children but overall, the prevalence of nephrocalcinosis ranges between 30 and 70% in the literature [6, 9, 44]. The median time span of 1.7 years between initiation of medical treatment and discovery of nephrocalcinosis in our pediatric cohort was comparable with the Norwegian cohort [9]. Our finding that one third of adults developed nephrocalcinosis is in agreement with a recent report by Chesher et al. [43]. Three out of five adult patients with nephrocalcinosis had hypercalciuria the year before or at time of the nephrocalcinosis diagnosis. The absence of hypercalciuria in some children with nephrocalcinosis may be because they were treated with burosumab at that time. Previous studies have questioned the accurateness of the urinary calcium/creatinine ratio to establish hypercalciuria in children [21]. Interestingly, we found that the age at start of XLH-related medication was inversely correlated with the occurrence of nephrocalcinosis and positively correlated with age at diagnosis of nephrocalcinosis. These findings, together with the lower prevalence in adults and the higher prevalence in children who start treatment before the age of 2 years, suggest that children who start treatment at an early age are more susceptible to develop nephrocalcinosis. This phenomenon has been proposed by others and our findings confirm this relationship [9, 46].

Hyperparathyroidism is common in XLH patients and thought to be the result of stimulation of parathyroid cells by FGF23 and by oral phosphate supplementation but could also be related to vitamin D deficiency [6, 47, 48]. In our cohort, 24% of the pediatric patients and 21% of the adult patients had hyperparathyroidism at inclusion, which is in line with the prevalence reported by Lecoq et al. [49]. About a third of the total population had developed hyperparathyroidism for a period of more than 6 months. In addition, almost 40% of adults with prolonged hyperparathyroidism developed hypercalcemia, indicating primary or tertiary hyperparathyroidism, and over 20% required parathyroidectomy. Taken together, these results indicate that hyperparathyroidism is prevalent in XLH and reinforces the need for PTH monitoring.

This study has several limitations. ORPHOS-NED identifies potential study participants by approaching pediatric and adult endocrinologists and nephrologists. Consequently, most patients receive medical treatment and are treated in a tertiary center. This study design does not allow us yet to draw conclusions on the prevalence of XLH in the Netherlands or on the XLH phenotype of patients treated in non-academic hospitals. However, inclusion of (non-academic) hospitals is ongoing. In addition, most children in this study were included in the University Medical Center Groningen, which is the reference center for burosumab in children in the Netherlands. For this reason, the phenotype of the children in this study may be more severe than of other pediatric XLH patients in the Netherlands who are not on burosumab treatment. Moreover, so far we collected and analyzed retrospective data, so there was some missing data and fasting status was often unknown. However, by carefully studying both paper and electronic medical records of all included patients in all participating hospitals, we were able to collect most of the data points. Lastly, it is important to note that all laboratory data at time of inclusion were collected irrespective of treatment with burosumab, phosphate and/or active vitamin D supplementation, which will affect TmP/GFR, serum phosphate and 1,25(OH)_2_D concentrations.

In conclusion, this nationwide cohort of adult and pediatric XLH patients enables us to study manifestations and complications of XLH. Our data show that adiposity, bone deformities, osteoarthritis, hearing loss, nephrocalcinosis, and hyperparathyroidism are highly prevalent in Dutch XLH patients. Fractures occur in a minority of patients, mainly in legs and feet. Moreover, this study confirms that early start of XLH-related treatment is beneficial for longitudinal growth but may pose a risk factor for development of nephrocalcinosis.
